# Brazil's heavy metal pollution harms humans and ecosystems

**DOI:** 10.1016/j.soh.2023.100019

**Published:** 2023-07-10

**Authors:** Joel Henrique Ellwanger, José Artur Bogo Chies

**Affiliations:** Laboratory of Immunobiology and Immunogenetics, Postgraduate Program in Genetics and Molecular Biology (PPGBM), Department of Genetics, Universidade Federal do Rio Grande do Sul (UFRGS), Porto Alegre, Rio Grande do Sul, 91501-970, Brazil

**Keywords:** Amazon, Brazil, Environmental health, Heavy metals, Pollution

## Abstract

This letter draws attention to the worrying situation of heavy metal pollution in Brazil, especially concerning the Amazon's Indigenous peoples affected by mercury contamination from illegal gold mining activities. Heavy metal pollution is also an emerging problem in other Brazilian biomes besides the Amazon Forest (e.g., Pampa biome in southern Brazil), as well as in coastal ecosystems/regions and large cities. Despite being a neglected problem, Brazil's heavy metal pollution causes significant detrimental impacts on human health and ecosystems. Finally, some alternatives to overcome this problem are suggested.

Brazil is a global leader in agricultural production [1] and has a diversified industry structure, being currently the largest economy in South America and the 12th largest in the world [[2], [3], [4]]. Moreover, Brazil is a mega-biodiverse country, with six terrestrial biomes (namely Amazon, Caatinga, Cerrado, Pantanal, Atlantic Forest and Pampa), an extensive marine coastline, and harbors large freshwater reserves [3]. Brazil also has significant natural reserves of ores with industrial importance, including those providing aluminum, copper, chromium, tin, iron, manganese, niobium, nickel, vanadium, zinc, and gold [5]. However, Brazil faces many problems related to social inequality and flaws in environmental protection [3]. This scenario not only allows, but facilitates, the pollution of Brazilian biomes with heavy metals (also known as “potentially toxic elements”) [6] from different sources. A body of evidence shows that vehicle pollution [7], industrial waste [8], mining activities [9], use and production of pesticides [10,11], among other activities [12,13], contribute to heavy metal pollution in Brazil.

Pollution of terrestrial and aquatic ecosystems with heavy metals has multiple effects on non-human animals, impairing nervous system, reproduction, behavior, antioxidant defense, among other functions [14,15]. A myriad of different organisms is impacted by such pollutants. Studies performed in Brazil evidenced that heavy metal exposure affects birds [16,17], mammals [18,19], reptiles [20,21], amphibians [22], and fish [20,23]. In addition to impacts on animal groups, heavy metal pollution harms human populations, especially through environmental exposure and food (e.g., mercury-contaminated fish) [15,23].

In Brazil, the Amazon region is being called the “new Minamata” [24,25], in reference to the Japanese city significantly affected by mercury pollution in the 1950s. This tragic event caused several human cases of neurological and developmental diseases due to consumption of fish and shellfish contaminated with mercury (methyl mercury - MeHg) released into Minamata Bay by a chemical factory that manufactured acetaldehyde and vinyl compounds. The Minamata mercury poisoning outbreak gave rise to the term “Minamata disease”, currently used to designate neurological cases of MeHg poisoning [26,27]. Mercury poisoning, due to the high MeHg neurotoxicity, may cause olfactory, visual and gustatory disturbances, psychiatric disorders, hearing and speech impairment, movement problems, among other neurological issues [26,27]. In addition to cases of acute mercury poisoning, residents living in Minamata Bay were chronically exposed to low-dose MeHg for about 20 years. Of note, chronic mercury poisoning triggers a wide range of somatosensory disorders due to diffuse damage to the somatosensory cortex [27]. In Brazil, Indigenous peoples are currently suffering from similar health problems due to mercury contamination linked to illegal gold mining activities in the Amazon [28,29], which increased 495% in Indigenous Lands from 2010 to 2020 [30].

A robust body of evidence supports that Amazonian communities are chronically exposed to mercury [31,32]. Basta et al. [28] performed a study in Brazilian Munduruku Indigenous villages (*Sawré Muybu*, *Poxo Muybu* and *Sawré Aboy*) and found mercury levels (based on 197 hair samples) ranging from 1.4 to 23.9 μg/g, with a 57.9% prevalence of mercury exposure ≥6.0 μg/g. The authors observed that chronic exposure to mercury affected especially women of childbearing age, being associated with high blood pressure [28]. Also studying Brazilian Munduruku Indigenous peoples, Oliveira et al. [29] found that individuals with MeHg exposure level ≥10 μg/g showed two-fold higher chances of cognitive deficits. In the same study, two cases of cerebellar ataxia were observed in individual with high MeHg exposure, and mercury-related impairment in the verbal fluency was also reported [29]. Other Brazilian Indigenous and riverine groups, such as Yanomami and Tapajós communities, are also exposed to mercury pollution due to gold mining activities [33,34].

In brief, neurotoxicity is the major issue associated with mercury exposure. In this sense, Fig. 1 exemplifies some mercury-related neurobehavioral outcomes observed in Amazonian human communities, based on data from Passos and Mergler [35]. However, mercury-related health issues are not limited to the nervous system. A recent study performed in the Brazilian Amazon has revealed that exposure to mercury increases the risk of anemia in children [36]. Chronic exposure to heavy metals is also related to genetic damage and molecular alterations linked to cancer and chronic diseases [37].

**Fig. 1:**
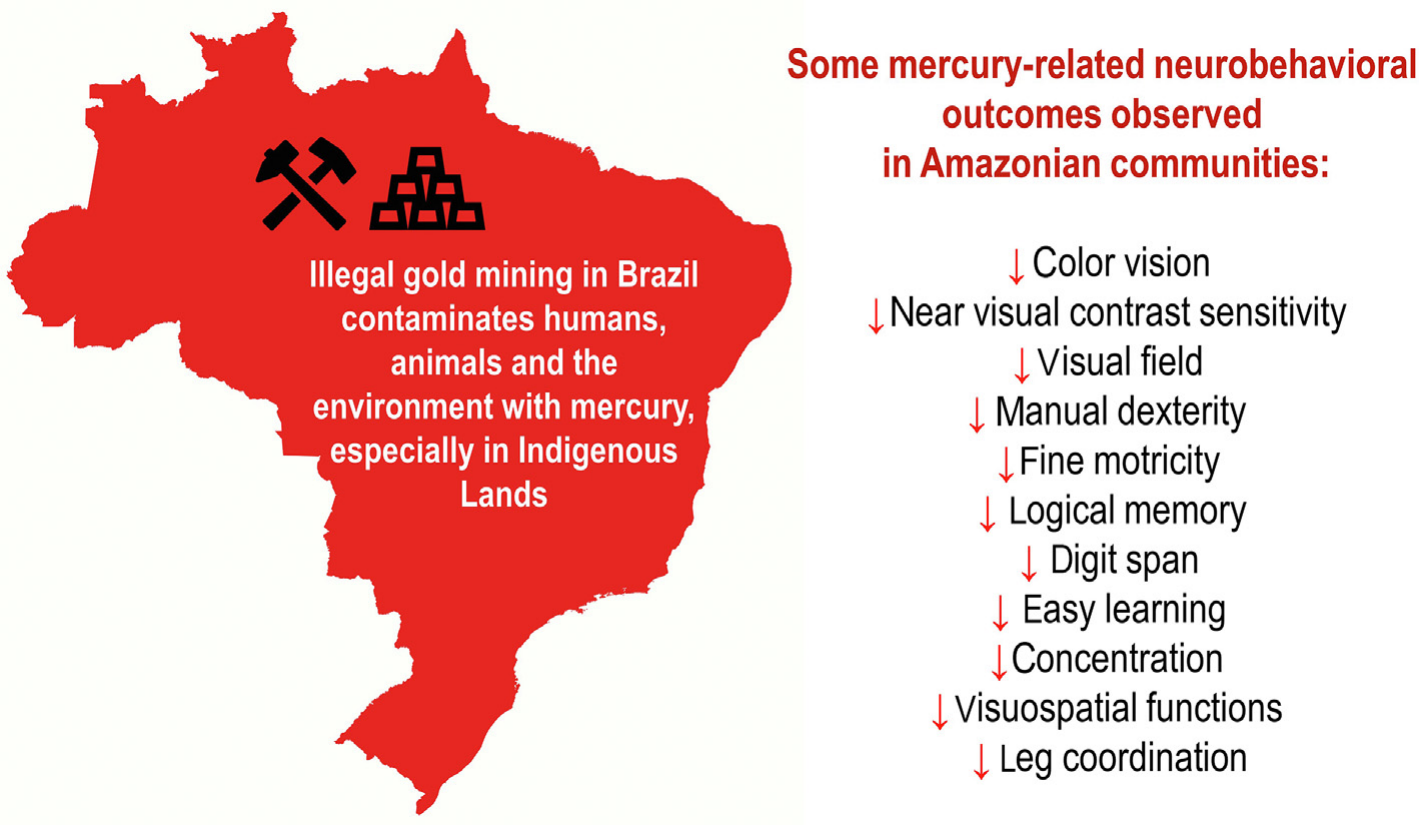
Brazil's current scenario of mercury contamination. Mercury-related neurobehavioral outcomes (in humans) based on Passos and Mergler [35]; only statistically significant results are shown. Figure created with Microsoft 365. Brazil's map obtained from MapChart (https://www.mapchart.net/).

Beyond the similarity between the symptoms of mercury poisoning, the route of mercury exposure and economic interests create a parallel between the communities of Minamata Bay and the Amazon region. In both communities, humans were exposed to mercury primarily through consumption of local food sources (i.e., mercury-contaminated fish). Furthermore, in both cases socio-economic interests (i.e., industrial production in Minamata and gold mining activities in the Amazon) associated with huge environmental neglect allowed the entry of massive amounts of mercury into the environment and food webs.

Although the control of illegal mining has been significantly retaken under Lula's administration [38], some Brazilian political groups act constantly to weaken the environmental protection of Indigenous Lands [39], and the deleterious effects of mercury on the environment and human health will be long-lasting. In Brazil, heavy metal pollution is not limited to the Amazon region. Some human and animal populations from Rio Grande do Sul, the southernmost Brazilian state, show high levels of heavy metals, potentially due to the inappropriate use of pesticides in this region economically dependent on cash crop plantations [[40], [41], [42]]. Also, the presence of heavy metals in Brazilian metropolises [43] and beaches [44] threatens humans and ecosystems. These pieces of evidence show that problems caused by heavy metal contamination affect different Brazilian biomes in addition to the Amazon Forest, including Atlantic Forest and Pampa biomes [42,43].

Studies based on environmental samples also evidenced heavy metal contamination of ecosystems in Caatinga, Cerrado, and Pantanal due to multiple anthropogenic activities (e.g., pesticide use, mining and industrial activities, urbanization, wildfires) [[45], [46], [47], [48], [49], [50], [51]]. Heavy metal pollution in a given biome may have more extensive impacts than anticipated. For example, Brazilian biomes have been suffering from intense wildfires (many of anthropogenic origin) [52] and ashes containing heavy metals can be transported over enormous distances, affecting the health of people living even at long distances from pollution sites [48]. Moreover, the rainforest can intercept atmospheric mercury residuals from gold mining activities and then accumulate this metal in soil and biomass. The forest-accumulated mercury may be released into the atmosphere again by anthropic activities such as wildfires and deforestation, thus potentially spreading and amplifying the deleterious impacts of this heavy metal on ecosystems [53,54]. In brief, heavy metal pollution is a problem that affects all Brazilian biomes and regions to some extent.

Brazil's heavy metal pollution should receive greater attention, along with biodiversity loss and deforestation. Environmental protection agencies must work to ban illegal mining in Brazil and control the entry of illegal mercury into the country, especially considering that Brazil is committed to the UN Minamata Convention on Mercury [55]. The international community can contribute by monitoring these actions and stop buying precious metals with suspicious origin. Taking under consideration the complex political and socioeconomic aspects that fuel gold mining in Brazil, the control of conflicts in Indigenous lands, as well as the reduction of social inequalities are fundamental to reduce illegal gold mining activities in the Amazon region [54]. Brazil also needs to advance its solid waste disposal policy [56], avoiding soil contamination with heavy metal-containing waste (e.g., e-waste, batteries), and improve the monitoring of heavy metal levels in human, animal, and environmental samples, based on the One Health perspective. Above all, actions must be coordinated and continuous, or we will be leaving a quite injurious long-lasting heritage to the world.

## Authors’ contributions (CRediT author statement)

JHE: Conceptualization, Investigation, Writing - Original Draft, Visualization. JABC: Writing - Review & Editing, Supervision.

## Funding

JHE receives a postdoctoral fellowship from *Coordenação de Aperfeiçoamento de Pessoal de Nível Superior* - CAPES (*Programa Nacional de Pós-Doutorado* - PNPD, Brazil). JABC receives a research fellowship from *Conselho Nacional de Desenvolvimento Científico e Tecnológico* - CNPq (*Bolsa de Produtividade em Pesquisa - Nível 1A*, CNPq, Brazil) and has research funded by 10.13039/501100002322*Coordenação de Aperfeiçoamento de Pessoal de Nível Superior* - CAPES (AUXPE 686/2020, Brazil).

## Conflicts of interest

The authors declare no conflicts of interests.
